# Intellectual-disability-associated mutations in the ceramide transport protein gene *CERT1* lead to aberrant function and subcellular distribution

**DOI:** 10.1016/j.jbc.2021.101338

**Published:** 2021-10-22

**Authors:** Norito Tamura, Shota Sakai, Loreto Martorell, Roser Colomé, Aya Mizuike, Asako Goto, Juan Darío Ortigoza-Escobar, Kentaro Hanada

**Affiliations:** 1 Department of Biochemistry & Cell Biology, National Institute of Infectious Diseases, Shinjuku-ku, Tokyo, Japan; 2 Molecular Genetics Department, Hospital Sant Joan de Déu, Barcelona, Spain; 3 Neuropsychology Unit, Pediatric Neurology Department, Hospital Sant Joan de Déu, Barcelona, Spain; 4 Movement Disorders Unit, Pediatric Neurology Department, Hospital Sant Joan de Déu, Barcelona, Spain; 5 Centro de Investigación Biomédica en Red de Enfermedades Raras - CIBERER-ISCIII, Madrid, Spain; 6 European Reference Network for Rare Neurological Diseases (ERN-RND), Tübingen, Germany

**Keywords:** ceramide transport protein, phosphorylation, intellectual disability, sphingomyelin, lipidome, Cas9, CRISPR-associated protein 9, CERT, ceramide transport protein, CRISPR, clustered regularly interspaced short palindromic repeat, ER, endoplasmic reticulum, FFAT, two phenylalanines in an acidic tract, GalCer, galactosylceramide, GlcCer, glucosylceramide, GM130, Golgi matrix protein 130, HA, hemagglutinin, hGH, human growth hormone, HRP, horseradish peroxidase, ID, intellectual disability, KI, knockin, KO, knockout, MLPA, multiplex ligation-dependent probe amplification analysis, mVenus, monomeric Venus, PH, pleckstrin homology, PtdIns(4)P, phosphatidylinositol 4-monophosphate, SD, standard deviation, SEM, standard error of the mean, SM, sphingomyelin, SRM, serine-repeat motif, START, steroidogenic acute regulatory protein-related lipid transfer, SV40, simian virus 40, VAP, vesicle-associated membrane protein-associated protein

## Abstract

The lipid molecule ceramide is transported from the endoplasmic reticulum to the Golgi apparatus for sphingomyelin production *via* the ceramide transport protein (CERT), encoded by *CERT1*. Hyperphosphorylation of CERT’s serine-repeat motif (SRM) decreases its functionality. Some forms of inherited intellectual disability (ID) have been associated with a serine-to-leucine substitution in the SRM (S132L mutation) and a glycine-to-arginine substitution outside the SRM (G243R mutation) in CERT; however, it is unclear if mutations outside the SRM disrupt the control of CERT functionality. In the current investigation, we identified a new CERT1 variant (dupAA) in a patient with mild ID that resulted from a frameshift at the *C*-terminus of *CERT1*. However, familial analysis revealed that the dupAA variant was not associated with ID, allowing us to utilize it as a disease-matched negative control for *CERT1* variants that are associated with ID. Biochemical analysis showed that G243R and S132L, but not dupAA, impair SRM hyperphosphorylation and render the CERT variants excessively active. Additionally, both S132L and G243R mutations but not dupAA caused the proteins to be distributed in a punctate subcellular manner. On the basis of these findings, we infer that the majority of ID-associated CERT variants may impair SRM phosphorylation-dependent repression, resulting in an increase in sphingomyelin production concurrent with CERT subcellular redistribution.

Intellectual disability (ID) is a developmental disorder that includes intellectual and adaptive functioning deficits in conceptual, social, and practical domains (1). ID has an overall general prevalence in the population of approximately 1%, which varies by age (1). Although the origins of ID are complex and diverse, specifying the origin of ID is crucial for developing rational intervention methods to ameliorate disorders. By using whole-exon sequencing analysis, several independent studies recently showed that *de novo* mutations in *CERT1* are associated with ID (Fig. 1*A*) (2, 3, 4, 5, 6, 7). *CERT1* encodes the ceramide transport protein CERT, which mediates interorganelle trafficking of ceramide from the endoplasmic reticulum (ER) to the *trans*-Golgi regions for the synthesis of sphingomyelin (SM) in mammalian cells (8, 9, 10). When a serine-repeat motif (SRM) of CERT undergoes multiple phosphorylations, the function of CERT is repressed (11, 12, 13). Among ID-related variants in *CERT1*, many, but not all, variants are mapped to the region encoding the SRM (2, 3, 4, 5, 6, 7). A few ID-associated variants in the CERT SRM have been experimentally shown to impair the SRM phosphorylation-dependent repression mechanism (7). However, whether ID-associated variants located at non-SRM regions affect the function of CERT remains unknown.

**Figure 1:**
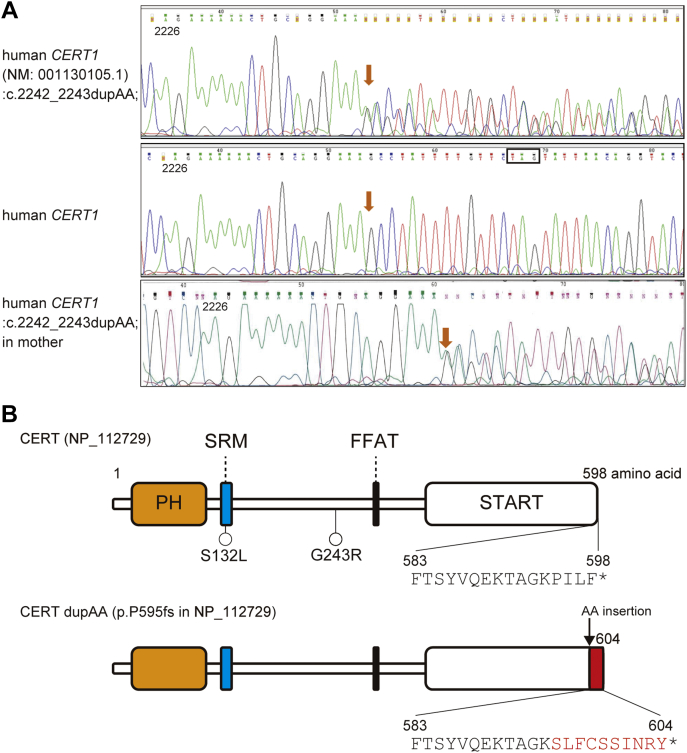
**A *CERT1* mutation found in an ID patient.***A*, the *CERT1* (NM: 001130105.1; *COL4A3BP*):c.2242_2243dupAA; p.(Pro749fs) mutation verified by Sanger sequencing. The *upper panel* shows the sequencing map of the proband (*orange arrow* indicates the exact position of the variant). The *middle panel* shows the normal sequence. The mother (*lower panel*) and the proband show the same change located at 2242 to 2243 bases, resulting in a truncating frameshift variant. *B*, distribution of ID-associated variants of the CERT structure (NP_112729), which includes a PH domain (*orange*), SRM (*blue*), FFAT motif (*black*), and a START domain (*white*). The terminal 4 amino acid residues (PILF) in WT CERT are changed to ten amino residues (SLFCSSINRY, marked by *red*) by the dupAA mutation. The *asterisk* represents amino acid termination.

In this study, we found a novel, although noncausative, mutation in *CERT1* in a patient with ID. We demonstrated, using comparative analyses of wild-type, ID-associated, and noncausative CERT variants, that various CERT variants with ID-associated mutations frequently impair SRM phosphorylation-dependent repression, resulting in an increase in SM synthesis concurrent with CERT subcellular redistribution.

## Results

### Phenotype of a proband

The proband is a 9-year-old boy who was born to a nonconsanguineous Moroccan family. He was referred for evaluation of ID to the Hospital Sant Joan de Déu, Barcelona, Spain. He presented with psychomotor developmental delay at 2 years old. At that age, he walked without support and he presented with speech delay with a limited number of words and no phrasing. He had a behavioral disorder that frequently shifted from one activity to another and exhibited recurrent mannerisms, such as repeatedly putting his hand to his mouth. In contrast, socialization skills and eye contact were normal.

He was born at term by an uneventful cesarean section and weighed 5000 g. His neonatal period was normal. His family history was unremarkable, except for one younger sister with developmental delay.

On physical examination at 9 years old, the vital signs were normal. Facial dysmorphism was not present. He had obesity (weight: 61.9 kg, >99th percentile, height: 140 cm, 73rd percentile) and macrocephaly (cranial circumference: 57.5 cm, >99th percentile, 3.17 standard deviations [SDs]). Abdominal, cardiac, and pulmonary examinations were unremarkable. The patient was intellectually impaired with good eye contact. He was able to engage in a conversation with diminished vocabulary. He showed repetitive behavior (stereotypies) with emotions. The remainder of his neurological examination (cranial nerves, motor and sensory function, and reflexes) was normal.

He was cognitively delayed and could not independently perform activities of daily living. At 9 years old, he was able to run, jump, and go up and down stairs. He ate using his hands and he required help with dressing and bathing. He named classmates from his classroom. He verbalized common words and two- or three-word phrases. He identified and wrote his name and surname. He knew how to count to 19. The Wechsler Intelligence Scale for Children – Fifth Edition showed a full scale composite score of 55, which was consistent with a mild ID (Table S1). However, he did not meet criteria for autism spectrum disorder.

Metabolic tests, including measurement of serum ammonia and lactate levels, thyroid and liver function tests, blood gas analysis, serum amino acid chromatography, MS/MS, and the urine organic acid profile were applied. However, all results were within normal limits. Similarly, video electroencephalography, auditory-evoked potentials, and brain MRI were normal, while the visual evoked potential showed a delay in nerve conduction through the optical pathways (P2 wave at 136 ms, amplitude of 15–20 μV).

### Heterozygous mutation in *CERT1* in the proband

Conventional karyotype analysis, multiplex ligation-dependent probe amplification analysis (MLPA) for ID, Prader–Willi methylation testing, and X-fragile analysis were normal. Array comparative genomic hybridization detected an approximately 493 Kb duplication of chromosome band 15q11.2 (22.756.505–23.249.681) and an approximately 127 Kb deletion of chromosome band 8p11.22 (39.258.894–39.386.158). These were not considered to cause the patient’s neurological symptoms. The TruSight One Expanded Sequencing Panel (Illumina) detected a *COL4A3BP* variant (NM: 001130105.1 [*COL4A3BP*]: c.2242_2243dupAA; p.[Pro749fs]). Other variants of interest that were not considered to cause the patient’s ID were also detected (Table S2).

The Gene Nomenclature Committee of the Human Genome Organization revised the official symbol of the gene from *COL4A3BP* to *CERT1* in 2019 (https://www.genenames.org/data/gene-symbol-report/#!/hgnc_id/HGNC:2205). Additionally, in most human tissue types, the predominant transcript of *CERT1* encodes a ceramide transport protein, CERT, of 598 amino acid residues (7). Therefore, we hereafter use the amino acid numbering of CERT for the *CERT1* gene product (Fig. 1*A*). The frameshift dupAA variant changes the terminal four amino acid residues (PILF) in wild-type (WT) CERT to ten amino residues (SLFCSSINRY) (Fig. 1*B*).

Although the heterozygous variant was confirmed by Sanger sequencing (Fig. 1, *A* and *B*), we concluded that it was noncausative of ID because it was segregated from ID in familial analysis. We came to this conclusion because the mother has the same variant, although she has no ID (Fig. 1*A*). By contrast, a sister of the patient does not have the variant although she was diagnosed with ID. The father does not have the variant (data not shown). To validate the effect of the dupAA mutation on the function of CERT, we established dupAA mutation knockin (KI) HCT116 cell lines (Fig. S1). In line with the Sanger sequence analysis, heterozygous and homozygous KI cell lines did not show any defects in CERT function in biochemical studies (Fig. S1). Therefore, we confirmed that the dupAA mutation was not causative in this patient with ID. While the dupAA was not associated with ID, we considered that this CERT variant could serve as an invaluable negative control to ID-associated CERT variants.

### Biochemical comparison of the CERT dupAA variant with ID-associated CERT variants

Phosphorylation of multiple serine/threonine residues of the CERT SRM downregulates the activity of CERT (11, 13). Serine-to-alanine substitution at the 132nd position (S132A) in CERT was shown to render the protein constitutively active (11), although the S132A variant has not been found in humans to date. However, human whole-exon sequencing studies have recently shown various examples of missense mutations (*e.g.*, S132L, S135C, S135P, and S138C) that generate amino acid replacements in the SRM of CERT and are associated with inherited ID disorders (2, 3, 4, 5, 6, 7). Amino acids that are affected by the ID-associated *CERT1* variants are highly conserved in model organisms (Fig. S2). In addition to serine residues in the SRM of CERT, glycine residue for the ID-associated G243R substitution, which is located in a non-SRM region, is also conserved, except for in *Caenorhabditis elegans* (Fig. S2). However, whether ID-associated variants located in non-SRM regions in CERT affect the phospho-state of the SRM, the activity of CERT, and/or subcellular localization remain unknown. Therefore, we decided to compare biochemical and cell biological features of S132L (which was expected to impair SRM phosphorylation) and G243R. To achieve this aim, we also used two controls, including WT CERT and a novel, but ID-unrelated variant, CERT dupAA.

**Figure 2:**
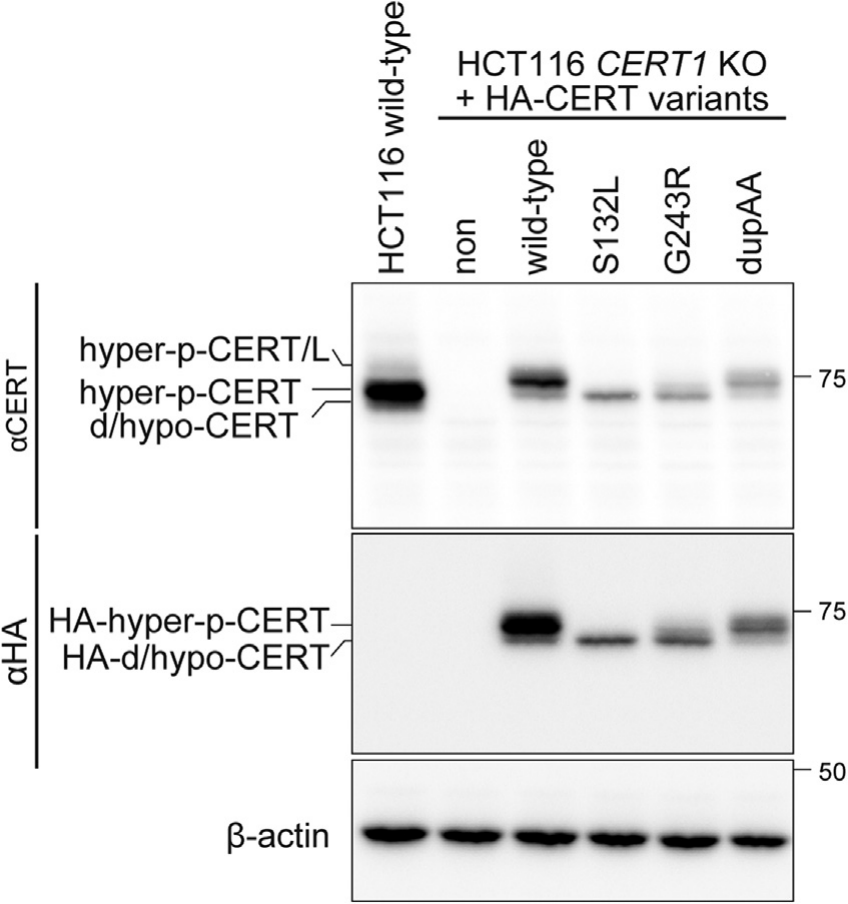
**S132L and G243R, but not the dupAA mutation, affect the phosphorylation status of CERT.** WT (HCT116 WT) and *CERT1* KO HCT116 cells that stably expressed various HA-CERT constructs (A) were analyzed by western blotting. Hyperphosphorylated (hyper-p) and de/hypophosphorylated (d/hypo-p) CERT (NP_112729.1) and a long isoform of CERT (CERT/L) (NP_005704.1), which is encoded by a splicing transcript variant of *CERT1* (7), are shown.

**Figure 3:**
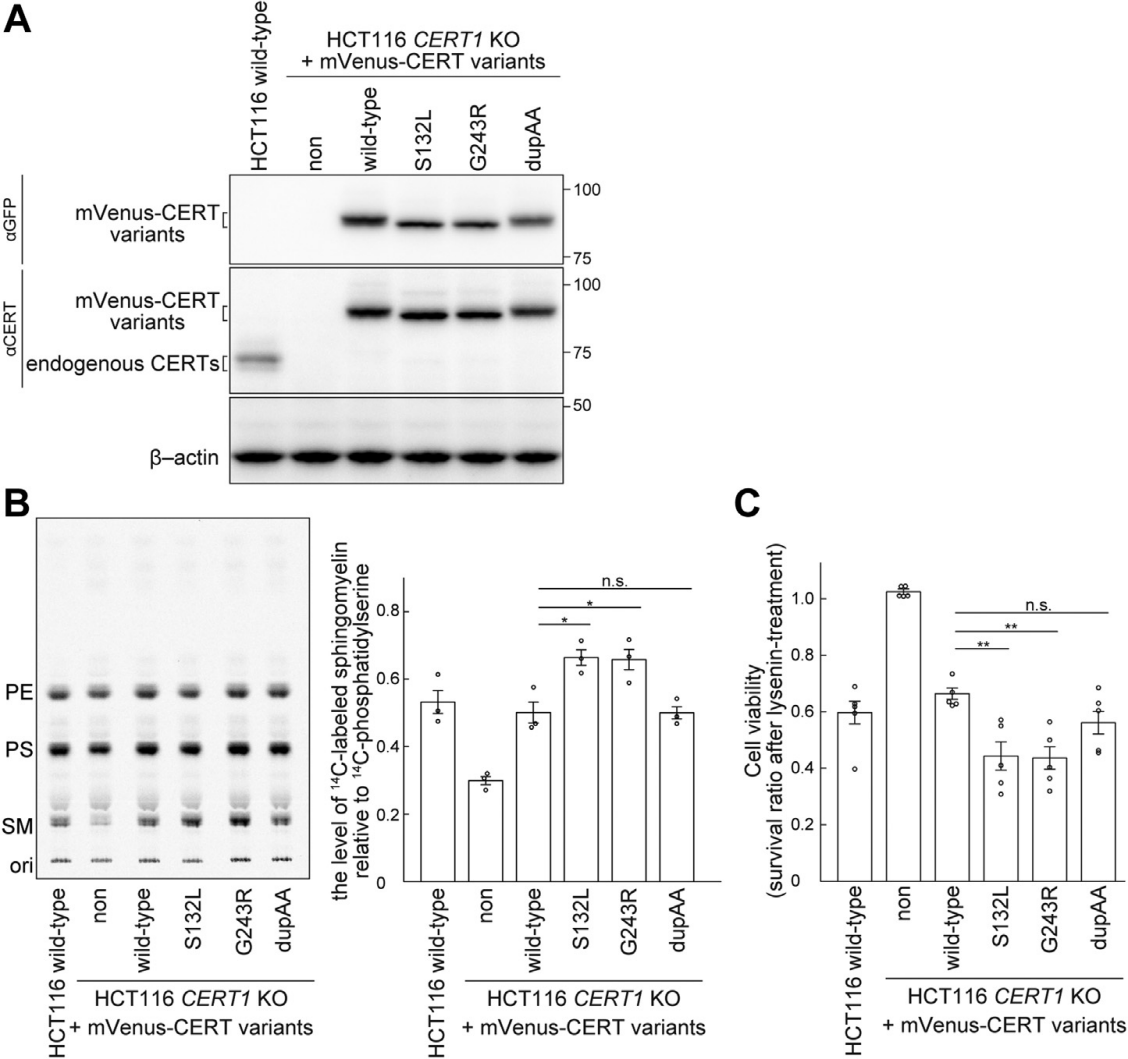
**S132L and G243R variants abnormally enhance the function of CERT.***A*, WT and *CERT1* KO HCT116 cells that stably expressed various mVenus-CERT constructs were analyzed by western blotting. *B*, WT and *CERT1* KO HCT116 cells that stably expressed various mVenus-CERT constructs were cultured with L-[U-^14^C]serine for 16 h. Metabolically labeled lipids that were separated on a TLC plate were visualized (representative image, *left*) and labeled SM was quantified (*right*). Data show mean ± SEM; n = 3 (∗*p* < 0.05; n.s., not significant). *C*, WT and *CERT1* KO HCT116 cells that stably expressed various mVenus-CERT constructs were cultured with 250 ng/ml of lysenin for 1 h. Cellular viability was measured with a lactate dehydrogenase cytotoxicity assay. Data show mean ± SEM; n = 5 (∗∗*p* < 0.01; n.s., not significant). PE, phosphatidylethanolamine; PS, phosphatidylserine.

To characterize CERT mutants in the absence of endogenous CERT, we used a HCT116 *CERT1* knockout (KO) cell line, in which both alleles of *CERT1* were disrupted in human colon-cancer-derived HCT116 cells. Various constructs encoding CERT mutants tagged with influenza hemagglutinin (HA) or monomeric Venus (mVenus) were stably expressed in the HCT116 *CERT1* KO cells and analyzed. Similar to the endogenous CERT in HCT116 cells, the ectopically expressed HA- or mVenus-tagged WT CERT showed a major hyperphosphorylated form, while the ectopically expressed HA- or mVenus-tagged CERT S132L only displayed de/hypophosphorylated forms (Figs. 2 and 3*A*). These findings are in line with our previous study on CERT S132A (11). Intriguingly, the marked shift from hyperphosphorylated to de/hypophosphorylated forms was also observed for the ectopically expressed HA- or mVenus-tagged CERT G243R, but not CERT dupAA (Figs. 2 and 3*A*). We then assessed the activity of CERT by metabolic labeling of SM with radioactive serine. Labeling of SM, but not other sphingolipids [*i.e.*, ceramide and glucosylceramide (GlcCer)] or glycerophospholipids (*i.e.*, phosphatidylserine and phosphatidylethanolamine), was reduced in *CERT1* KO cells compared with parental HCT116 cells. This reduction was restored to parental control levels when mVenus-tagged WT CERT was expressed in *CERT1* KO cells (Fig. 3*B*). When the mutant CERT S132L or G243R constructs were expressed, the levels of labeled SM were significantly higher than those observed in the WT CERT rescued cells (*p*-value, S132L-0.016, and G243R-0.020) (Fig. 3*B*). This finding occurred despite almost equal or lower expression levels of these CERT mutants compared with WT CERT levels (Fig. 3*A*). By contrast, expression of the mutant CERT dupAA restored SM synthesis, but not beyond the WT control level (Fig. 3*B*). This finding indicated that CERT dupAA was functional for ER-to-Golgi trafficking of ceramide. The sensitivity to lysenin, which is an SM-binding cytolysin, was monitored as another measure of SM synthesis (8, 14). Cells that expressed the CERT S132L and G243R mutants, but not the dupAA mutant, showed higher lysenin sensitivity compared with WT CERT expressing cells (*p*-value, S132L-0.006, and G243R-0.004) (Fig. 3*C*). This finding suggested that the intracellular CERT S132L and G243R mutants, but not the dupAA mutant, showed higher activity than WT CERT.

### Lipidome of cells expressing CERT variants

**Figure 4:**
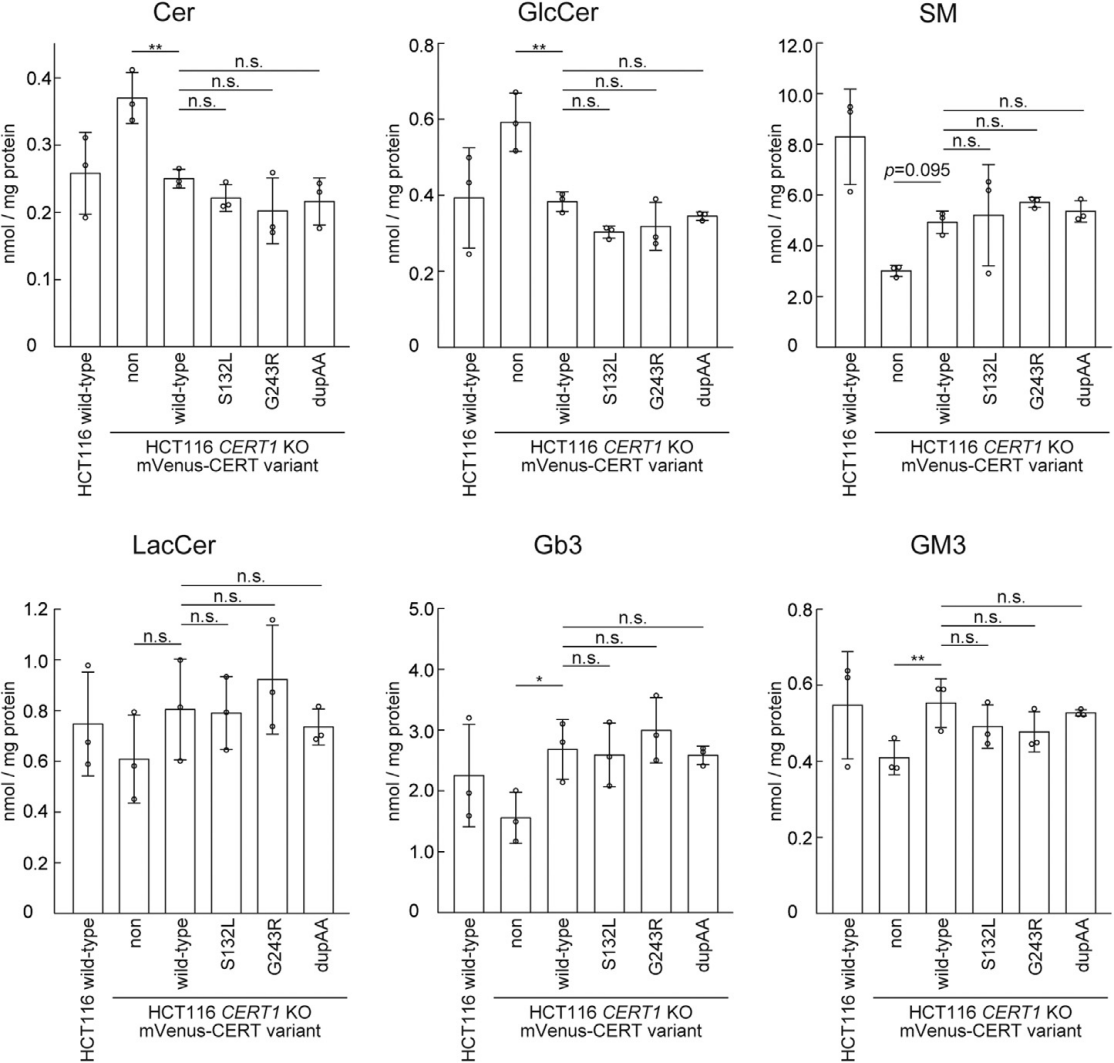
**Lipidome in WT and *CERT1* KO HCT116 cells stably expressing various mVenus-CERT constructs.** The amount of various sphingolipid types in cells was quantified by LC-MS/MS. Data show mean ± SD; n = 3 (∗*p* < 0.05; n.s., not significant). Cer, ceramide; Gb3, Gb3 globoside; GlcCer, glucosylceramide; GM3, GM3 ganglioside; LacCer; lactosylceramide; SM, sphingomyelin.

To compare sphingolipid levels between each variant ectopically expressing *CERT1* KO cells, we performed lipidomic analysis of the whole cell. When compared with the WT cell control, not only the amount of ceramide but also that of GlcCer increased in *CERT1* KO cells (Fig. 4), possibly due to compensatory responses to the decrease in the amount of SM, in agreement with a previous study (15). These alterations were abolished by WT CERT ectopic expression in *CERT1* KO cells (Fig. 4). However, under our experimental condition, when the ID-associated variants S132L and G243R were ectopically expressed in *CERT1* KO cells, there was no significant difference in sphingolipid levels in the variant-expressing cells compared with the WT CERT-expressing cells (Fig. 4). Therefore, the sphingolipid composition of the whole cell did not change, even though abnormally activated CERT variants were expressed.

### Punctate distribution of ID-associated CERT variants, but not the dupAA variant, in cells

**Figure 5:**
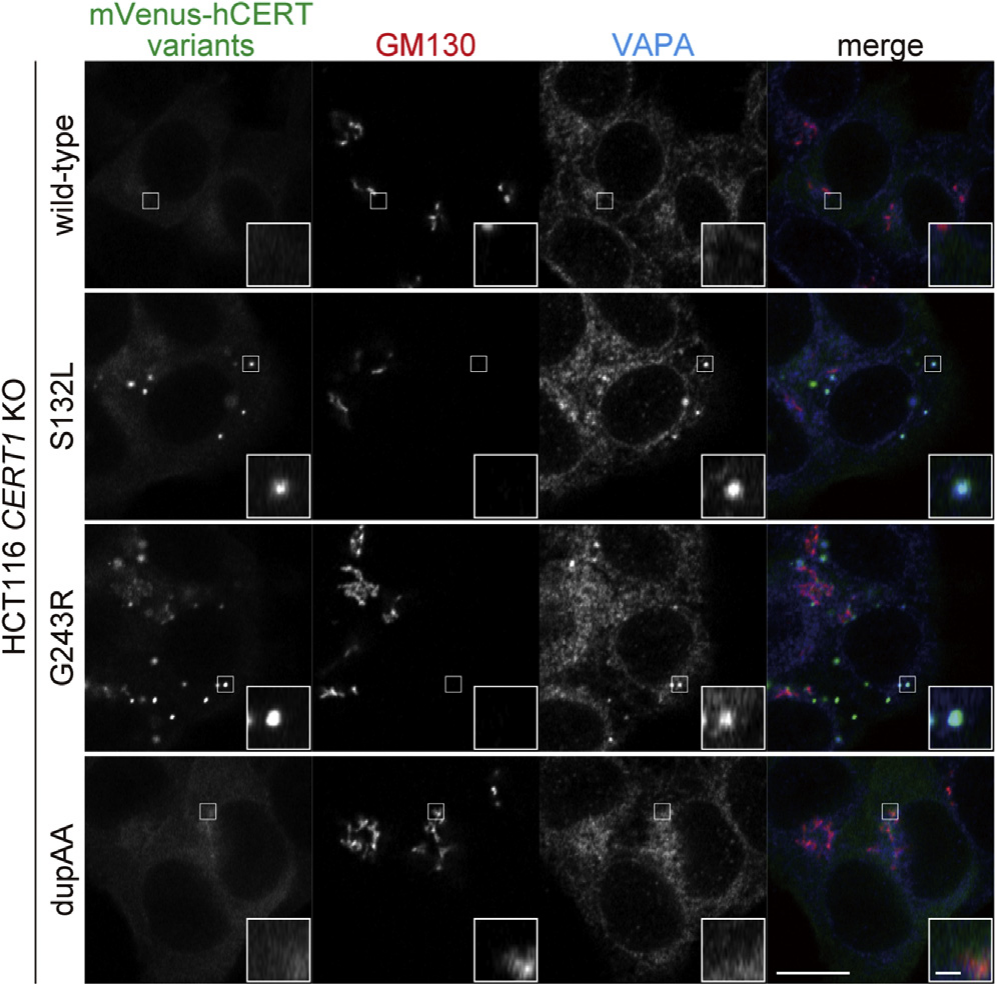
**ID-associated mutations induce punctate subcellular distribution of CERT.***CERT1* KO HCT116 cells that stably expressed the indicated mVenus-CERT constructs were analyzed by immunofluorescence microscopy. The data are representative of two independent experiments. Areas enclosed by squares are enlarged (*insets*). Scale bars, 10 μm and 1 μm (*inset*).

We recently showed that ID-associated S135P and S135C substitutions in CERT caused these proteins to become abnormally active and induce a punctate subcellular distribution (7). When mVenus-tagged CERT variants were expressed in HCT116 *CERT1* KO cells, WT CERT was diffused throughout the cytoplasm with partial enrichment in the perinuclear regions, which are adjacent to a 130-kDa *cis*-Golgi matrix protein (GM130) (Fig. 5). The CERT S132L and G243R mutants showed a punctate distribution pattern, and part of the puncta was costained with the vesicle-associated membrane protein-associated protein (VAP)-A (Fig. 5). By contrast, CERT dupAA showed no punctate distribution and showed a pattern that was similar to that of CERT WT (Fig. 5). These results taken together with our previous studies showing analogous behavior of other activated CERT mutants (*i.e.*, S132A, S135A, S135C, S135P, and S315E), which showed a punctate distribution (7, 11, 16), led us to conclude that most constitutively activated CERT mutants commonly display a subcellular punctate distribution. This conclusion supports our previous proposal that the intracellular punctate distribution pattern of CERT mutants may be applicable as a molecular diagnostic assay to assess whether CERT is abnormally activated by *CERT1* mutations.

## Discussion

Clinical genetic sequencing is frequently used to diagnose individuals with neurodevelopmental disorders, including ID. A recent meta-analysis and systematic review of the diagnostic performance of clinical sequencing through next-generation sequencing showed a diagnostic yield of 28.2% for ID (17). Estimates of diagnostic yield vary considerably across individual studies, ranging from 8% to 61% (18, 19). In the case of our proband, all of the genetic tests that were accessible to us were conducted without a successful outcome. Therefore, the causative alteration of his ID remains unknown. The dupAA variant was originally discovered during analysis of our patient with ID using a sequencing panel targeting disease-associated regions of the exome. However, the mutation was found to be irrelevant to ID in familial analysis and showed no phenotypes in our analysis (Fig. S1).

Our recent study showed that ID-associated missense mutations at the codon for serine 135 in *CERT1* abrogated SRM phosphorylation-dependent repression of CERT activity (7). In the present study, we found that the G243R variant, which is an ID-associated mutation that occurs outside of the SRM (3, 7), was also defective in hyperphosphorylation of the SRM. This resulted in expression of abnormally activated CERT (Fig. 3) and punctate subcellular distribution was observed (Fig. 5), similar to the ID-associated CERT SRM variants S132L (this study) and S135P (7). Moreover, CERT dupAA, which is a “disease-matched” but ID-noncausative CERT variant with activity to support the synthesis of SM, was found to retain a hyperphosphorylated SRM under normal culture conditions and to not display a punctate distribution, similar to the WT CERT (Figs. 3 and 5). On the basis of these results together with a previous study (7), we propose that most CERT variants with ID-associated mutations have common biochemical and cell biological outcomes.

CERT has a motif [known as two phenylalanines in an acidic tract (FFAT) motif] that binds to VAP and a pleckstrin homology (PH) domain that preferentially binds to phosphatidylinositol 4-monophosphate [PtdIns(4)P] (8, 20). As previously established (7, 11, 16), highly active CERT mutants are distributed to not only perinuclear Golgi areas (costained with the *cis*-Golgi marker GM130), but also to punctate structures. Interestingly, punctate structures linked to CERT are costained with VAP but not with GM130. Additionally, distribution of CERT to punctate structures needs the PtdIns(4)P-binding ability of the CERT PH domain (7, 11, 16). Although the exact nature of the punctate structures is unknown at the moment, they may represent membrane contact sites between VAP-enriched ER subregions and *trans*-Golgi areas, where CERT effectively facilitates ceramide transport from the ER to the Golgi. Alternatively, the punctate structures could represent “emergent” contacts between VAP-enriched ER subregions and PtdIns(4)P-rich non-Golgi compartments, rerouting ceramide to endosome/lysosome compartments and avoiding excessive SM synthesis in the Golgi complex even when CERT is abnormally activated. As a recent study has shown that overexpression of VAP family proteins results in the formation of stacked membrane arrays in the ER (21), it is possible that CERT-dependent clustering of VAP in the ER results in the formation of stacked membrane arrays. Additional research will be required to understand the punctate structure entity.

Among ID-associated mutations, how the G243R variant shows deficiency in hyperphosphorylation of the SRM is unknown. In cells, the predominant form of CERT is likely to be a homotrimer (22). Hyperphosphorylation of the SRM might generate an intramolecular (or intra-CERT trimer) electrostatic interaction that induces conformational changes in CERT to mask the PH and steroidogenic acute regulatory protein-related lipid transfer (START) domains (11, 12). However, which region electrostatically interacts with the phosphorylated SRM in CERT remains unclear. The possibility that G243R interfered with a tentative electrostatic interaction is unlikely because, if this were the case, the G243R variant would have shown an abnormally activated phenotype, even with a hyperphosphorylated SRM. We cannot exclude the possibility that G243R itself did not directly affect hyperphosphorylation of the SRM, but that G243R-dependent impairment of a conformational change resulted in easy access of protein phosphatases to the SRM. This could then indirectly facilitate dephosphorylation of the SRM. To test these possibilities, the precise structure of the full-sized CERT and/or its oligomer should be resolved. High-resolution structures of the isolated PH domain (12, 23, 24) and START domain (25, 26, 27) of CERT have been determined.

Human *CERT1* produces at least three splicing variants of transcripts (7): The *CERT1* transcript isoform encoding the 598-amino acid protein CERT [also known as Goodpasture antigen-binding protein (GPBP)Δ26)] is ubiquitously expressed in human tissues, and the isoform encoding a 624-amino acid protein named CERT/L (also known as GPBP), which has an extra 26-amino acids prior to the START domain of CERT, is also widely but less abundantly expressed in various tissues. The third isoform (here hereafter referred to as isoform 3) is predicted to encode a 752-amino acid protein with a 128-amino acid extra fragment prior to the initial methionine of CERT/L, but its transcription is absent or rare except for sperm. The present study analyzed mutated constructs of CERT, but not of CERT/L, but previous studies showed that CERT/L as well as CERT mediates ER-to-Golgi trafficking of ceramide in cells (7, 8). Revert *et al.* have reported that CERT/L, but not CERT, is exported from cells *via* a nonconventional secretory pathway (22, 28). Revert *et al.* (28) also reported another GPBP-related 91-kDa protein that was proposed to be translated from a noncanonical ACG codon, whereas the *CERT1* transcript isoform 3 has an ATG codon in the 5′-distal region (NCBI Reference Sequence: NP_001123577.1). It is unclear whether the ID-associated mutations in *CERT1* affect features of extracellular GPBP and/or their putative membrane-bound derivatives.

ID-associated mutations abnormally enhanced *de novo* synthesis of SM in *CERT1* KO cells ectopically expressing the CERT variants (Fig. 3). However, the whole-cell lipid composition, even SM, of these cells did not change among variants (Fig. 4). The failure to detect significant changes in the lipidome may be due to interference with external sphingolipids present in the culture medium. We could not culture HCT116 cells in a serum-free medium. To overcome this issue, we will need to use other cell lines capable of growing in a serum-free medium. Another, but mutually nonexclusive, possibility is that ID-associated CERT variants have a dramatic effect only in a specific cell type. A previous study showed that a patient with ID had a delay in myelination in the cerebrum (7). Galactosylceramide (GalCer) and its derivative sulfatide comprise the major sphingolipid class in the myelin sheath, and they play an important role in electrophysiology of the myelinated nerve (29, 30). Because ceramide is the common precursor for SM and GalCer (31), abnormally activated CERT, which delivers ceramide preferentially to the site of SM synthesis, might destroy an appropriate balance between SM and GalCer in the myelin sheath. To address these questions, further investigations using a more specific cell line, such as glial cells (oligodendrocytes in the central nervous system and Schwann cells in the peripheral nervous system) with ID-associated mutations, are required.

## Experimental procedures

### Mutation screening of the patient with ID and assessment

#### Samples of the patient and family

Biological samples from all family members were collected with the appropriated signed informed consent. Genomic DNA was prepared from peripheral blood leukocytes using standard procedures (32).

#### Determination of CGG repeat size in the *FMR1* gene

PCR analysis was performed to measure normal alleles, and Southern blotting was performed for large expansions as previously described (33).

#### MLPA for ID

The presence of deletions or duplications of ID-related genes was evaluated by MLPA, using the commercially available Salsa Probemix P036-E1 and P070-B1 (MRC Holland) according to the manufacturer’s standard protocol and reagents. The amplification products were separated by capillary electrophoresis using an ABI3500 Genetic Analyzer (Applied Biosystems), and data were analyzed in detail with Coffalyser software (MRC Holland).

#### Prader–Willi molecular testing

Prader–Willi molecular testing was performed using MLPA (SALSA kit ME028-B2 PW/AS; MRC Holland). This kit contains specific probes for the 15q11-q13 region (TUBGCP5, NIPA1, MKRN3, MAGEL2, NDN, SNRPN, UBE3A, ATP10A, and GABRB3), the critical region (APBA2), and control regions. MS-MLPA was used to detect changes in copy number (large deletions, imprinting center deletions) and DNA methylation.

#### Comparative genomic hybridization

This analysis was carried out using a comparative genomic hybridization oligonucleotide microarray (human genome Cytoarray Plus 180K; Agilent Technologies) that was distributed throughout the entire genome (*qChip Post*). Samples were hybridized against an internal reference DNA of the same sex. The data were analyzed with Genomic Workbench 7.0 software (Agilent Technologies) and interpreted using specified parameters as follows: Algorithm ADM2 ≥6.0; abs (log2ratio) ≥0.25; probes ≥3.

#### Next-generation sequencing

Clinical exome sequencing was carried out using the TruSight One Expanded Sequencing Panel (Illumina) according to the manufacturer’s instructions. This panel covers 6713 disease-associated genes. As a whole, library preparation was performed using Illumina kits, and sequencing was carried out in the MiSeq Illumina platform. Bioinformatics analyses were performed for variant calling and interpretation enriching with fundamental databases, such as GnomAD, ExAC, and ClinVar.

#### Sanger sequencing

Sanger sequencing and PCR amplification were performed according to standard procedures. Sequencing was performed using the Big Dye Termination cycle sequencing kit 3.1 (Applied Biosystems) by following the manufacturer’s standard protocol and sequenced on an ABI 3500 genetic analyzer. ABI SEQSCAPE software version 2.5 (Applied Biosystems) was used to perform sequence analysis.

### Cell culture

Human colon-cancer-derived HCT116 cells (RIKEN Cell Bank) were cultured in low-glucose Dulbecco’s modified Eagle’s medium (WAKO) supplemented with 10% fetal bovine serum in a 5% CO_2_ incubator.

### Plasmids, antibodies, and reagents

cDNAs encoding the full-length human CERT (NP_112729) and its variants were amplified by PCR and subcloned into pMXs-IB together with the human influenza HA tag or the mVenus tag. We used pSpCas9 (BB)-2A-GFP (pX458) (Addgene plasmid # 48138) for establishment of the KI cell line. For western blotting analysis, rabbit polyclonal anti-CERT (ab72536; 1:500, Abcam), rat monoclonal anti-HA conjugated with horseradish peroxidase (HRP; clone 3F10; 1:5000, Roche), rabbit polyclonal anti-GFP (598; MBL, 1:5000), and mouse monoclonal anti-β-actin (sc-47778; 1:5000, Santa Cruz) antibodies were used as primary antibodies. Anti-mouse (NA934; 1:10,000, Cytiva) and anti-rabbit (172-1011; 1:10,000, Bio-Rad) HRP-conjugated IgG were used as secondary antibodies. For immunostaining, mouse monoclonal anti-GM130 (610822; 1:200, BD Bioscience) and rabbit polyclonal anti-VAPA (HPA009174; 1:100, Sigma-Aldrich) were used as primary antibodies. AlexaFluor 594-conjugated anti-mouse IgG (A11032; 1:1000) and AlexaFluor 647-conjugated anti-rabbit IgG (A32795; 1:1,000, Thermo Fisher Scientific) were used as secondary antibodies. Lysenin was a gift from Dr Sekizawa (Zenyaku Kogyo, Tokyo).

### Establishment of gene-edited cell lines

*CERT1* KO HCT116 cells were generated as described previously (7). In KI cell lines, CRISPR guide RNA sequences, which were designed for human *CERT1* genes, were cloned into the pX458 vector. The target sequence was 5′-GGCACCAGCCTCAGTGTTAA-3′. A donor sequence with or without the patient-derived mutation flanked by approximately 350-bp homology arms of *CERT1* was subcloned into the donor vector. This vector contains the simian virus 40 (SV40) PGK promoter, a human growth hormone (hGH) polyA signal, and a neomycin-resistant gene (Fig. S1). Each donor sequence was engineered to contain sufficient silent mutations to avoid recognition and cleavage by Cas9. HCT116 cells were cotransfected with the pX458 vector carrying the abovementioned guide RNA and the donor vector with a transfection reagent (ViaFect; Promega). After 48 h, GFP-positive cells were isolated using a cell sorter (BD FACSMelody; BD Biosciences). Gene-edited cells were selected by 10 μg/ml neomycin (Nacalai Tesque) and single clones were obtained. Heterozygous and homozygous clones with the desired gene insertion in *CERT1* alleles were identified by genomic PCR and DNA sequencing. Genomic PCR primers were 5′-GCTAATTGGTCCAATGTGAGG-3′ and 5′-GGTTGCAGTGAGCCGTGATGGTGTCATTG-3′.

### Retroviral preparation and establishment of stable cell lines

Stable cell lines were generated as described previously (7). Briefly, cells were transiently transfected using a transfection reagent (FuGENE6; Promega) with retroviral vectors. After 48 h, culture medium containing retrovirus was collected and filtered through a 0.45-μm syringe filter unit (Merck Millipore). Cells were incubated with retrovirus and 8 μg/ml polybrane (Sigma-Aldrich). Uninfected cells were removed by 2 μg/ml Blasticidin-S (Kaken Pharmaceutical Co, Ltd).

### Immunoblotting

Cells were lysed with a lysis buffer (50 mM Tris-HCl [pH 7.5], 150 mM NaCl, 1 mM EDTA, 1% Triton X-100, complete EDTA-free protease inhibitor cocktail [Roche], and phosphatase inhibitor cocktail 2 and 3 [Sigma-Aldrich]). After centrifugation at 20,000*g* for 20 min, the supernatant was collected and protein concentrations were adjusted by the bicinchoninic acid method (Thermo Fisher Scientific) using BSA as the standard. The lysates were solubilized with immunoblot sample buffer (46.7 mM Tris-HCl [pH 6.8], 5% glycerol, 1.67% SDS, 1.55% dithiothreitol, and 0.003% bromophenol blue). The immunoblot samples were separated by SDS-PAGE, transferred to an Immobilon-P polyvinylidene difluoride membrane (Millipore), and blotted with primary and secondary antibodies. Each protein signal was detected with Immobilon Western Chemiluminescent HRP substrate (Millipore). Signal intensities were captured using LuminoGraph (ATTO). The images were processed using Photoshop CS6 (Adobe).

### Sphingolipid analysis

Cells were seeded in a 6-cm dish at a density of 0.5 × 10^6^ cells/dish in 5 ml of culture medium. After 24 h, cells were washed twice with PBS and harvested. A total of 1 nmol each of the internal standards C17:0 SM, C17:0 ceramide (Cer), C17:0 lactosylceramide, C17:0 glucosylceramide, C17:0 Gb3, and d18:1-d5-C18:0 GM3 (Avanti Polar Lipids) were then added for LC-MS/MS analysis. The amount of protein was determined and lipids were extracted from the cells. Sphingolipids were analyzed by an LC-MS system that consisted of a Prominence UFLC system (Shimadzu) coupled to a 3200 QTRAP System (SCIEX) as described previously (27).

### Immunofluorescence

Cells that were grown on coverslips (Matsunami) were washed with PBS and fixed with Mildform 10N (Wako) for 15 min at room temperature. Fixed cells were permeabilized with 0.1% TritonX-100 in PBS for 5 min, blocked with 3% BSA (Sigma-Aldrich) in PBS, and incubated with specific primary antibodies for 1 h. After washing with PBS, cells were incubated with secondary antibodies for 1 h. The coverslips were observed using a confocal laser microscope (LSM700; Carl Zeiss) with a 100× oil-immersion objective lens (1.46 NA; Carl Zeiss) and analyzed using ZEN software (Carl Zeiss). For the final output, the images were processed using Photoshop CS6.

### Metabolic labeling with radioactive serine and TLC

Metabolic labeling of sphingolipids with radioactive serine was performed as described previously (7). Radioactive lipids on TLC plates (Millipore) were detected using an image analyzer (Typhoon FLA 7000; GE Healthcare). The intensities of radioactive lipid bands were determined by densitometric scanning using ImageJ software (National Institutes of Health). The relative intensity level of SM to phosphatidylserine was used as a measure of the relative amount of labeled SM.

### Lysenin tolerance assay

Cells were seeded at an initial density of 1.0 × 10^4^ cells/well in a 24-well plate and cultured in normal culture medium overnight. The cells were incubated in serum-free DMEM containing 250 ng/ml lysenin for 1 h. Cellular viability was measured using lactate dehydrogenase cytotoxicity assay kit (Nacalai Tesque) as described previously (7). Luminescence was measured by an iMark microplate reader (Bio-Rad).

### Statistical analysis

For data analysis of biochemical assays, one-way analysis of variance followed by Dunnett’s test or Tukey’s test were performed for multiple comparison testing. Descriptive statistics are presented as the mean ± standard error of the mean (SEM) or SD from technical replicates. Results were considered significant at the 95% significance level (*p* < 0.05). Statistical analysis was performed using R software (R Core Team).

### Study approval

Written informed consent was obtained from the patient’s parents. This study was approved by the Review Board and Ethics Committee of Hospital Sant Joan de Déu (approval number: ART-04-21) and followed the Declaration of Helsinki Ethical Principles for Medical Research Involving Human Subjects. The biochemical and cell biological experiments in this study were also approved by the Medical Research Ethics Committee of the National Institute of Infectious Diseases (approval number: 1062).

## Data availability

Unprocessed images and raw data for quantitative results have been posted at a public data repository (https://doi.org/10.6084/m9.figshare.7409891). All remaining data are contained within the article and supporting information.

## Supporting information

This article contains supporting information.

## Conflict of interest

The authors declare that they have no conflicts of interest with the contents of this article.
