# Supplementary material for: Intellectual-disability-associated mutations in the ceramide transport protein gene CERT1 lead to aberrant function and subcellular distribution
Source: J Biol Chem. 2021 Oct 22;297(5):101338. doi: 10.1016/j.jbc.2021.101338 (PMC8605338; doi:10.1016/j.jbc.2021.101338)
Supplement: Supplemental Figures S1, S2 and Tables S1, S2 [file mmc1.pdf]

Supporting Information to

Aberrant functional control and subcellular distribution of the ceramide transport protein by intellectual disability-associated mutations in *CERT1*

Norito Tamura, Shota Sakai, Loreto Martorell, Roser Colomé, Aya Mizuike, Asako Goto, Juan Darío Ortigoza-Escobar, and Kentaro Hanada

Supporting Information list

Figure S1. The dupAA mutation does not affect the function of CERT in the KI cells.

Figure S2. Sequence alignment of CERT proteins.

Table S1. Wechsler Intelligence Scale for Children - Fifth Edition (WISC-V).

Table S2. Proband variants.

Figure S1

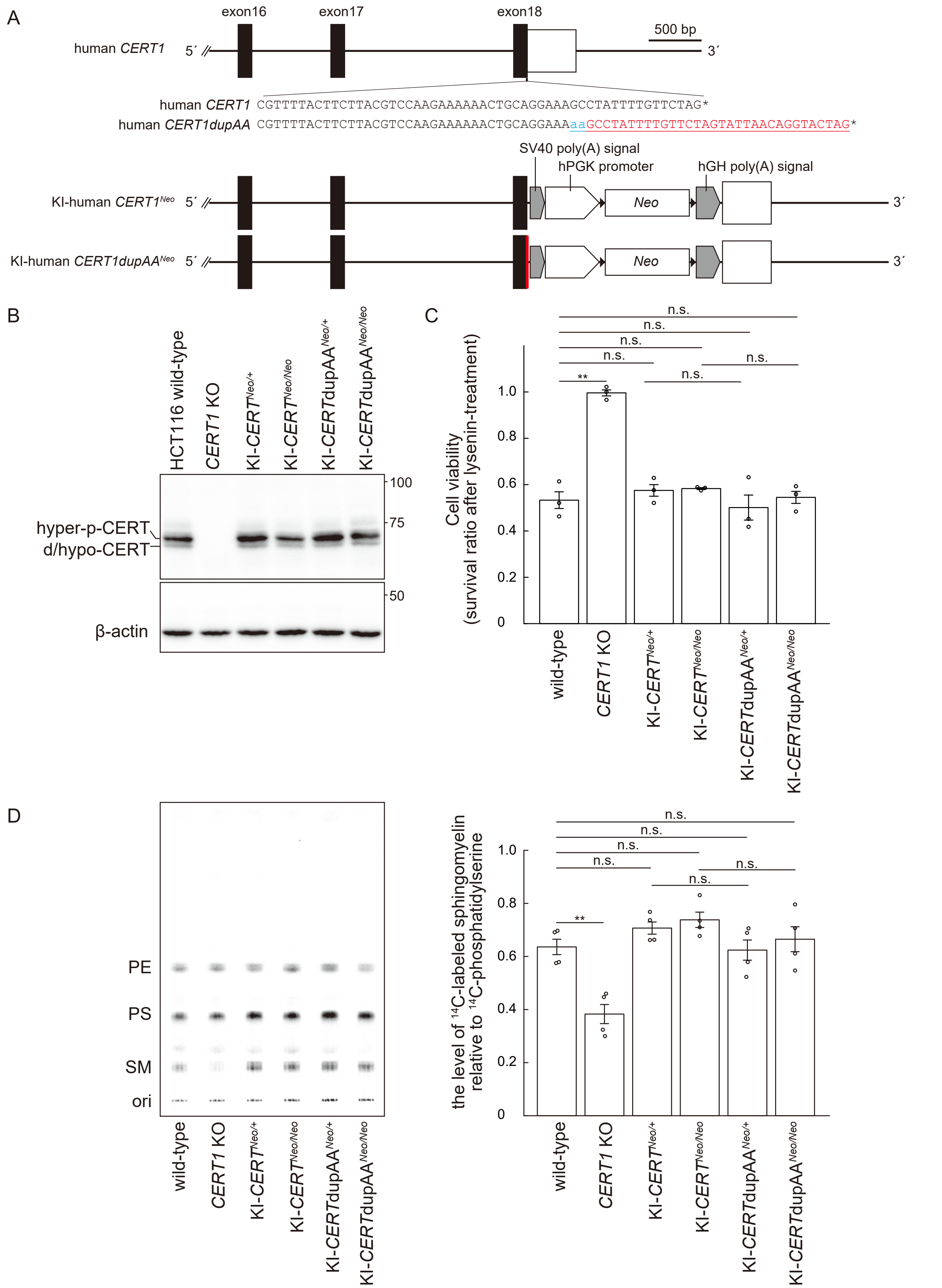

Figure S1, dupAA mutation does not affect to the function of CERT in the KI cells
(A) Schematic representation of the location of genome-edited sequences of human *CERT1*. The coding regions and 3′-UTR are shown in black and white rectangles, respectively. The red highlighted sequence (upper panel) and red line (lower panel) represent an extended sequence caused by a patient-derived mutation (blue). In the KI cells, edited gene sequences with desired gene insertions are shown (lower panel). *Neo*, neomycin-resistant gene.
(B) WT, *CERT1* KO, *CERT* KI, and *CERT*dupAA KI HCT116 cells were analyzed by western blotting. Heterozygous and homozygous KI-CERT mutants are shown as *Neo/+* or *Neo/Neo*, respectively. hyper-p-CERT, hyperphosphorylated CERT; d/hypo-CERT, de/hypophosphorylated CERT.
(C) WT, *CERT1* KO, *CERT* KI, and *CERT*dupAA KI HCT116 cells were cultured with 250 ng/ml lysenin for 1 h. Cellular viability was measured by the lactate dehydrogenase cytotoxicity assay. Data show mean ± SEM; n = 3 (** $p < 0.01$; n.s., not significant).
(D) WT, *CERT1* KO, *CERT* KI, and *CERT*dupAA KI HCT116 cells were cultured with L-[U-^{14}C]serine for 16 h. Metabolically labeled lipids separated on a TLC plate were visualized (representative image, left) and labeled SM was quantified (right). PE, phosphatidylethanolamine; PS, phosphatidylserine. Data show mean ± SEM; n = 4 (** $p < 0.01$; n.s., not significant).

Figure S2

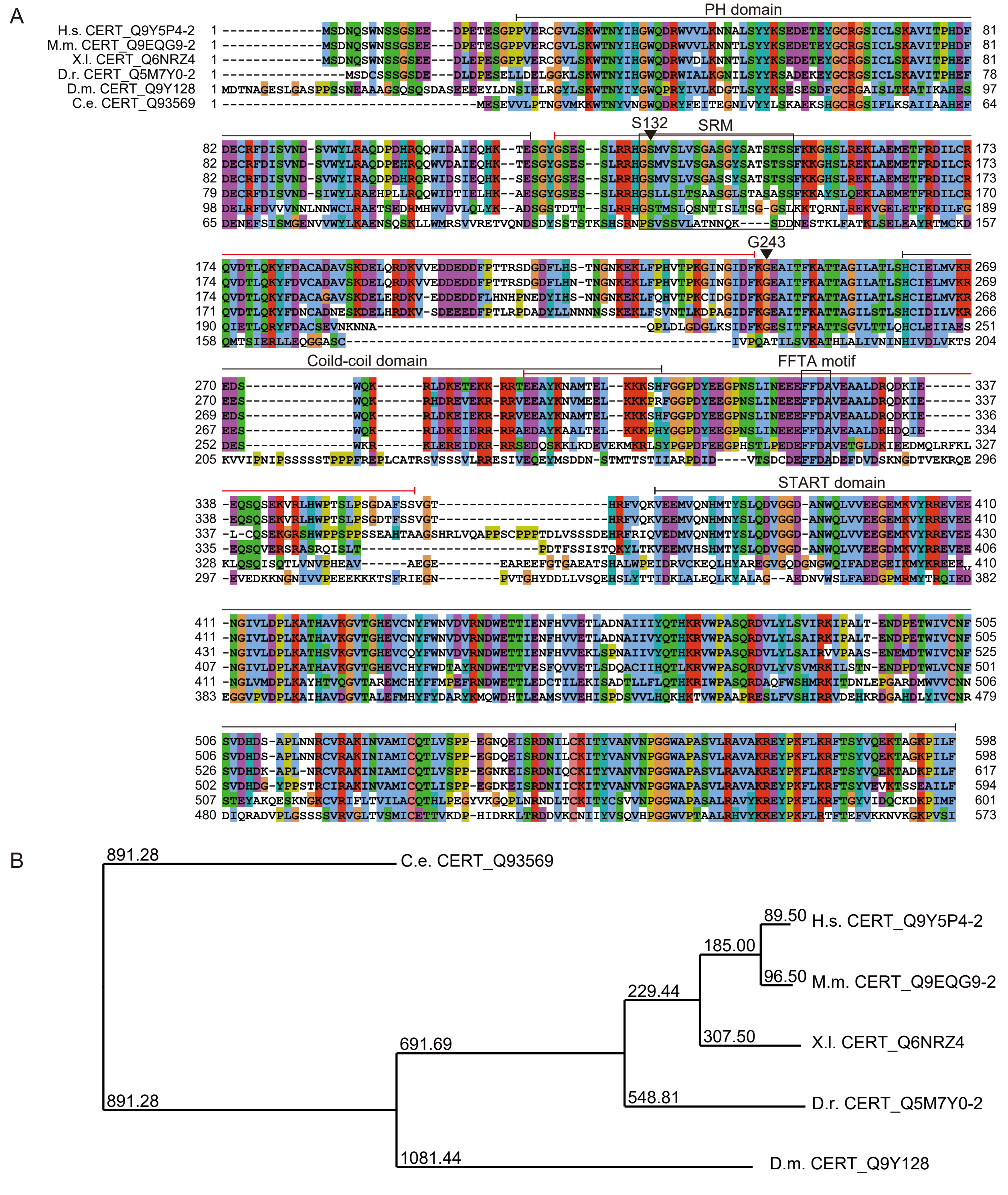

Figure S2, Sequence alignment of CERT proteins
(A) Comparison of the amino acid sequence of CERT in various organisms. *Homo sapiens* (H.s.) CERT (ProtUnitKB accession number: Q9Y5P4-2), *Mus musculus* (M.m.) CERT (Q9EQG9-2), *Xenopus laevis* (X.l.) CERT (Q6NRZ4), *Danio rerio* (D.r.) CERT (Q5M7Y0-2), *Drosophila melanogaster* (D.m.) CERT (Q9Y128), and *Caenorhabditis elegans* (C.e.) CERT (Q93569) are shown. Alignment was generated using CLUSTALW (http://www.genome.jp) and Jalview (http://www.jalview.org) software. Residues were colored using the clustalx color scheme implemented in Jalview. The black lines above the sequences represent the PH domain, coiled-coil domain, and START domain. The red lines above the sequences represent the disorder regions predicted by PSIPRED (http://bioinf.cs.ucl.ac.uk/psipred/). The black rectangles represent the SRM and FFAT motif. ID-associated mutations are indicated by black arrowheads.
(B) The Phylogenetic tree of CERT in various organisms was constructed by the neighbor joining tree method using Jalview software. Sequence similarities between each pair based on BLOSUM62 are shown.

Table S1

WISC-V index	Composite score	Percentile rank	95% confidence interval	Qualitative Intellectual Classification
Verbal comprehension (VCI)	45	<0.1	42-60	Extremely low
Visual Spatial (VSI)	67	1	63-77	Very low
Fluid Reasoning (FRI)	72	3	67-81	Very low
Working Memory (WMI)	51	0.1	47-63	Extremely low
Processing Speed (PSI)	60	0.4	56-74	Extremely low
Full Scale (FSIQ)	55	0.1	51-63	Extremely low

Table S1. Wechsler Intelligence Scale for Children - Fifth Edition (WISC-V)

WISC-V of the proband at 8 years and 2 months of age showed a mild intellectual disability (FISQ composite score of 55). The Visual Spatial Index (VSI) and Fluid Reasoning Index (FRI) were in the very low part of the Qualitative Intellectual Classification, while the Verbal Comprehension Index (VCI), Working Memory Index (WMI), and Processing Speed Index (PSI) were in the extremely low part.

Table S2

Gene	Location genomics	Protein/DNA	Genotype/ Matches the clinical phenotype	ACMG Classification	OMIM phenotype (#OMIM)/ inheritance
ABCA4 (NM_000350.2)	1-94473807	p.Gly1961Glu/c.5882G>A	Heterozygosity /No	Pathogenic; rs1800553	Stargardt disease 1 (# 248200), autosomal recessive
ANO5 (NM_001130105.1)	11-22257752	p.Gly231Val/c.692G>T	Heterozygosity /No	Pathogenic; rs137854523	Muscular dystrophy, limb-girdle, autosomal recessive 12 (# 611307), autosomal recessive
LTBP2 (NM_000428.2)	14-75018994	p.Pro432Leu/c.1295C>T	Heterozygosity /No	Pathogenic; rs201818754	Microspherophakia and/or megalocornea, with ectopia lentis and with or without secondary glaucoma (# 251750), autosomal recessive
ABCC6 (NM_001171.5)	16-16256935	p.Arg1141*/c.3421C>T	Heterozygosity /No	Pathogenic; rs72653706	Pseudoxanthoma elasticum, forme fruste (# 177850), autosomal dominant
XYLT1 (NM_022166.3)	16-17232391	c.1588-3C>T	Heterozygosity /No	Benign; rs201818754	Desbuquois dysplasia 2 (# 615777), autosomal recessive
KAT6A (NM_006766.4)	8-41834547	p.Ser448Gly/c.1342A>G	Heterozygosity /No	Benign; rs149459548	Arboleda-Tham syndrome (# 616268), autosomal dominant
KCNT1 (NM_020822.2)	9-138656997	p.Leu386Phe/c.1156C>T	Heterozygosity /No	Uncertain significance; rs780875110	Developmental and epileptic encephalopathy 14 (# 614959), autosomal dominant
WDR11 (NM_018117.11)	10-122663608	p.Tyr994Cys/c.2981A>G	Heterozygosity /No	Uncertain significance	Hypogonadotropic hypogonadism 14 with or without anosmia (# 614858), autosomal dominant
NALCN (NM_052867.2)	13-101881825	p.Ser515Arg/c.1545C>G	Heterozygosity /No	Likely pathogenic	Congenital contractures of the limbs and face, hypotonia, and developmental delay (# 616266), autosomal dominant
SETBP1 (NM_015559.2)	18-42533181	p.Asp1292Glu/c.3876C>G	Heterozygosity /No	Likely benign: rs139106261	Schinzel-Giedion midface retraction syndrome (# 269150), autosomal dominant
COL4A3BP (NM_001130105.1)	5-74675186	p.Pro749fs/ c.2242_2243dupAA	Heterozygosity /Yes	Pathogenic	Mental retardation, autosomal dominant 34, (# 616351), autosomal dominant

Proband variants

Other gene variants found in the proband exome are listed with information from the American College of Medical Genetics and Genomics (ACMG) Classification and the Online Mendelian Inheritance in Man (OMIM). *COL4A3BP* is an alternative name of *CERT1*.
